# Mineral Trioxide Aggregate Mixed with Normal Saline, Calcium Chloride or KY Jelly as Apical Plug in Simulated Open Apices: An *In vitro* Microleakage Study

**Published:** 2013-12-24

**Authors:** Payman Mehrvarzfar, Hengameh Akhavan, Sara Ghasemi, Fatemeh Khodaei, Sohrab Tour Savadkouhi, Omid Dianat

**Affiliations:** a *Department of Endodontics, Dental Branch, Islamic Azad University of Medical Sciences, Tehran, Iran; *; b *General Dentist, Private Practice, Tehran, Iran; *; c *Endodontist, Private Practice, Tehran, Iran; *; d *Iranian Center for Endodontic Research, Research Institute of Dental sciences, Department of Endodontics, Dental School, Shahid Beheshti University of Medical Sciences, Tehran, Iran*

**Keywords:** Apical Plug, Calcium Chloride, Dye Penetration, KY Jelly, Microleakage, Mineral Trioxide Aggregate, Open Apex

## Abstract

**Introduction:** Mineral trioxide aggregate (MTA) mixed with normal saline has short working time, delayed setting time, and poor consistency when used as an apical plug. A preliminary study suggested that substituting normal saline with KY Jelly or 5% calcium chloride (CaCl_2_) as a vehicle expedites the setting time of MTA. The present *in vitro* study compared the microleakage of ProRoot MTA mixed with normal saline (MS) to that of ProRoot MTA mixed with KY Jelly and/or 5% CaCl_2_ in simulated canals with open apices. **Materials and methods:** Thirty six single-rooted extracted human teeth were cleaned and shaped with ProTaper rotary system to make 36 standardized artificially created open apices. Teeth were randomly divided into three experimental groups (*n*=10) and two control groups (*n*=3). In group 1, MTA was mixed with normal saline (MS) and placed into the canals to form 4 to 5 mm apical plugs. In group 2, MTA was mixed with 5% CaCl_2_ (MC) and in group 3, MTA was mixed with KY Jelly (MK). The other two groups served as positive and negative controls. The remaining canal spaces in the experimental groups were backfilled with thermoplasticized gutta-percha without sealer. Dye penetration and clearing was used to evaluate the sealing ability of each group. The samples were then examined under stereomicroscope to measure the microleakage of different MTA mixtures in mm. Data were statistically analyzed using One-Sample Kolmogorov-Smirnov test for determination of normal distribution and then by one-way ANOVA and Tukey’s tests to detect any significance. **Results:** Positive and negative controls responded as expected. The MS group showed the least mean dye penetration value. There was a significant difference between MS with other groups (*P*<0.05) but no difference was found between MC and MK groups. **Conclusion:** Within the limitations of this *in vitro* study, we can conclude that among these three vehicles, normal saline mixed with ProRoot MTA has the least amount of microleakage in canals with open apices.

## Introduction

Sealing the apical part of immature open apex teeth in apexification procedure, is still one of the greatest challenges of endodontic treatments. The absence of a matrix against which the filling materials can be packed may lead to over-extrusion which can be a reason for treatment failure. Moreover, the shape and size of apical foramen and the absence of a proper sealing matrix causes apical microleakage which is one of the most important problems with endodontic treatment of immature teeth. Application of mineral trioxide aggregate (MTA) apical plug, to seal the open apex has proved to be a good solution [1, 2]. Although MTA has several advantages including favorable sealing in root-end filling and perforation repair, it has been suggested that its long setting time (~3 h) will result in increased possibility of bacterial leakage [3]. Several studies have shown a reduction in its setting time by addition of calcium chloride (CaCl_2_) or KY Jelly. It has been shown that mixing MTA with either NaOCl gel, KY Jelly or %5 CaCl_2_ is associated with a reduced setting time up to 20 to 25 min [4, 5].In a recent study, it was shown that addition of KY liquid or CaCl_2_ to MTA improved the handling properties and reduced the setting time of MTA [6].Other studies have shown higher biocompatibility of MTA mixed with either normal saline or KY Jelly and amalgam compared to the substances obtained from the resin-modified glass ionomer cement [7].In addition, osteoblast and fibroblast attachment and spreading patterns on MTA mixed with these additives was similar to MTA mixed with water [6]. This was the basis for the present study aiming to assess the apical microleakage of MTA mixed with CaCl_2_ and KY Jelly in simulated open-apex human teeth using an *in vitro* dye-penetration method.

## Materials and methods

Thirty six single-rooted mandibular or maxillary premolars, freshly extracted for periodontal reasons or orthodontic treatment plan were used. Periapical radiographs both in buccolingual and mesiodistal directions were taken. Inclusion criteria were the single-rooted teeth with straight canals with a root curve of ≤15 degrees, absence of fractures or microcracks, internal or external resorption or extensive intracanal calcifications. The specimens were initially cleaned under tab water by a brush to eliminate residual debris. They were then stored for 24 h in 0.5% sodium hypochlorite for decontamination. Then in order to improve the accessibility of the canals, dried specimens were cut from cement enamel junction (CEJ) using a diamond disk #0.3 (D&Z, Darmstadt, Germany) mounted on a handpiece under cooling water spray. To eliminate the apical ramifications and standardize the exit of the canals, a fissure bur #245 (D&Z, Darmstadt, Germany) was used to cut the apical 2 mm of the roots perpendicular to the long axis of the tooth. Working length (WL) was established 1 mm short of the length of the file tip while apically visible. The coronal two third of the canals were prepared using ProTaper S1 and SX files (Dentsply Maillefer, Ballaigues, Switzerland). Then, S1, S2, F1, F2 and F3 ProTaper files were used to the WL to complete the cleaning and shaping of the canals. Divergent shape of open apical foramen was prepared using Profile #40/.06 (Dentsply Maillefer, Ballaigues, Switzerland) in a retrograde manner. The roots were held in damp gauzes during the preparation processes and 5.25% sodium hypochlorite was used for irrigation. The prepared specimens were kept in 0.9 mL normal saline.

Specimens were placed into damp floral foam up to the CEJ level. A hole similar in size to a damp cotton pellet was excavated into the foam. A hand condenser #70 was inserted to the WL to assure the absence of residual foam or cotton in the canal. Canals were then dried with paper points and proper hand pluggers (Dentsply Maillefer, Ballaigues, Switzerland) were chosen for the placement of apical plugs. After all specimens were mounted into the foam and the preparation procedures were completed, specimens were randomly divided into three groups of ten (*n*=10) and two control groups of two.

***Group 1; MTA+Normal saline (MS): ***After being dried with paper cone, apices were obturated with ProRoot MTA (Dentsply, Tulsa Dental, Tulsa, OK, USA) with orthograde technique based on the instructions of the manufacturer. MTA was loaded in a messing gun (EndoGun, Medidenta Woodside, NY, USA) and then pushed into the apical third of the canal as far as possible using a plugger #4. When plugger was inserted 0.5 to 1 mm short of the WL, resistance was felt. At this point, no further pushing was applied and preferably a thick flat-cut-end paper cone damped with 0.9 mL normal saline was used to push the MTA plug further to WL. ProRoot MTA was applied incrementally and condensation was done for each layer applied. This procedure was carried out till the MTA plug reached ~4 mm of thickness. Paper cone was used to dry the canal and the rest of the canal was filled by thermoplasticized gutta-percha (Obtura II, Spartan/Obtura, Fenton, Missouri, USA) without sealer and the coronal part of the root was filled with dressing paste (Coltosol, Coltene, Switzerland) and packed.

***Group 2; MTA+CaCl***_2_ ***(MC): ***The same procedure was followed except for the application of the MTA mixed with 5% liquid CaCl_2_ (Merck, Darmstadt, Germany). According to the manufacture’s instruction, 0.3 mL of normal saline was mixed with 5% CaCl_2_ and 1 g MTA powder to prepare the sealing material.

***Group 3; MTA+KY Jelly (MK): ***The same procedure was followed except for the application of the mixture of MTA and KY Jelly (Johnson & Johnson, Midrand, South Africa). According to the manufacture’s recommendation, 1 g MTA powder was mixed with 0.25 mL gel to prepare the sealing material.

***Positive Control (C+): ***Three open-apex canals were cleaned and shaped just like the other three groups. Canals were obturated with single cone and tug back was assured. No apical plugs were applied.

***Negative Control (C-): ***Three open-apex canals were cleaned and shaped just like the other four groups. Glass Ionomer (Fuji II LC, GC America, Chicago, IL, USA) was used to seal the apical 4 mm of the roots and the whole root surface was covered with two layers of nail polish. Canals were obturated with single cone and presence tug back was assured. 

After this procedure, all specimens were placed in a closed container. Water was added to the container to keep the moist. The container was then placed into an incubator of 37^º^C under 100% humidity for 72 h so that the vapor simulates the oral circumstance.

After three days, all root surfaces except for the apical 2 mm were covered with sticky wax. Two layers of nail polish were then added on the same areas with a time interval of 3 h. The specimens were then placed in a black dye container vertically for a week in the room temperature. The teeth were then rinsed under the tap water for 5 min and dried. Layers of sticky wax and nail polish were removed from the root surfaces using a scalpel. Each specimen was then placed into 50 cc of 10% HCl for 24 h. After rinsing with tap water for 5 min to wash out the acid, the teeth were respectively placed in 70%, 80%, 90%, 96% and 100% ethylic alcohol for 5 h each, to become fixed. To complete the clearing process, specimens were first placed into 50% methyl salicylate solution for at least 3 h and then stored in 100% methyl salicylate solution up to the time of stereomicroscopic examination.

**Table 1:** Comparative apical microleakage values (mm) of the study groups

**Study groups**	**Microleakage mean (SD) **	**P** ^ a^	**P** ^ b^
**ProRoot MTA (MS)**	0.31 (0.21)	0.000	0.000
**MTA+%5 CaCl**_2_ ** (MC)**	4.58 (0.23)	-	0.97
**MTA+KY Jelly (MK)**	4.62 (0.49)	*-*	-

*a: Compared with MC; b: Compared with MK*

Linear technique was used in the present study to assess and score the leakage. Dye penetration amount in millimeter was measured under 16× magnification of the stereomicroscope (Olympus SZ X16, Hamburg, Germany) along the MTA plug of the transparent roots based on the microscope calibration.

Obtained data were statistically analyzed using one sample Kolmogorov-Smirnov test to determine the normal distribution and then by ANOVA and post-hoc Tukey’s tests to detect any significance. The confidence level of 95% (*α*=0.05) was considered for statistical evaluation.

## Results

Thirty six teeth were included in the present study. Positive control group (*n*=3) included 3 specimens which showed significant dye penetration. Negative control group (*n*=3) presented with no dye microleakage. Among specimens in three test groups, only one in the MS group did not show any microleakage.

**Figure 1:**
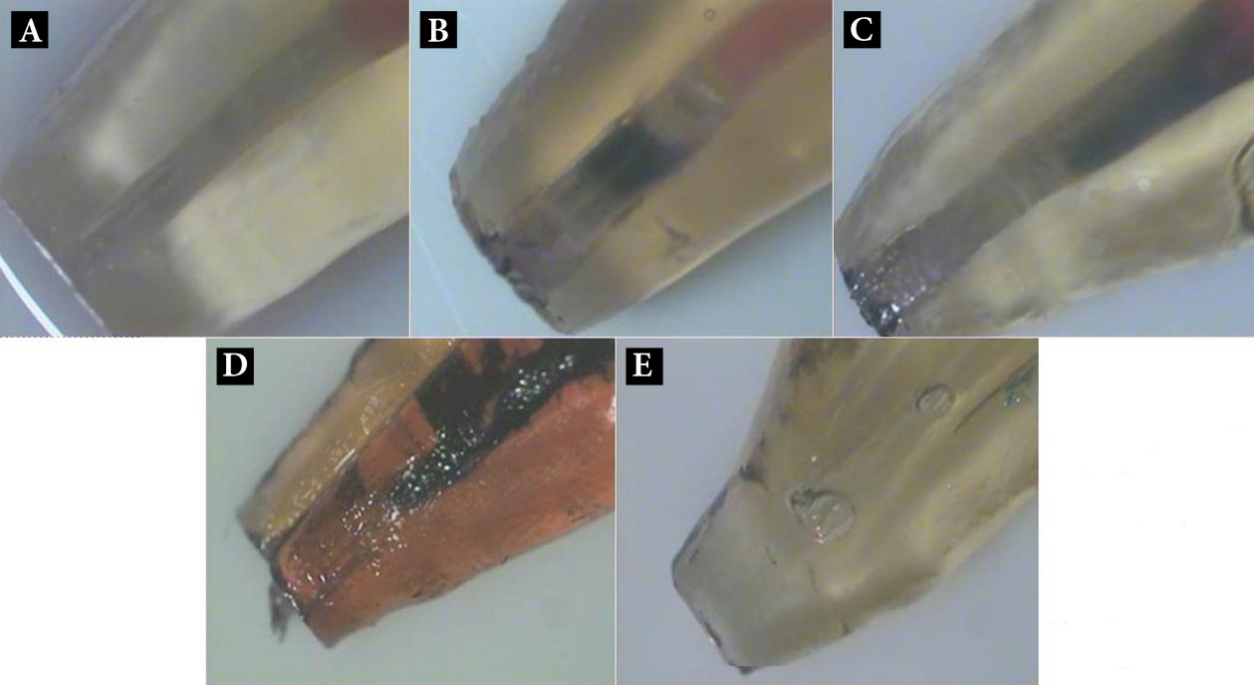
Microleakage evaluation of cleared samples in different groups; *A)* MTA mixed with normal saline; *B)* MTA mixed with CaCl_2_; *C)* MTA mixed with KY Jelly; *D)* Positive control; *E)* Negative control

One-sample Kolmogorov-Smirnov confirmed the normal distribution of the data. One-way ANOVA test revealed a significant difference between the microleakage values of MS, MC and MK groups (*P*<0.05) (Table 1). Tukey’s post-hoc test showed that the microleakage in the MS group is significantly less than that of MC and MK groups (*P*<0.05). However, the microleakage values of the two latter groups, were not significantly different (*P*>0.05 actual) (Figure 1).

## Discussion

In the present study the combination of MS provides a better sealing of the roots with open apices compared to the combination of ProRoot MTA and 5% CaCl_2_ (MC) or KY Jelly (MK). Also no statistically significant difference was found between MC and MK groups in terms of apical sealing.

According to the limitations of the present *in vitro* study, generalization of its results to the clinical situations should be done cautiously. In the experimental situation, apical plug could be easily standardized for all specimens, which is not the case for the clinical practice. Animal studies with the same design and materials are needed to confirm our results. However, this study may be used as a base for future *in vivo* studies.

Several studies have assessed characteristics of different MTA mixtures, including the biocompatibility of white MTA mixed with KY Jelly [7], setting time of the MTA mixed with either saline, 2% Lidocaine, 3% NaOCl gel, KY Jelly, 2% CaCl_2_ and 5% CaCl_2_ [5], the compressive strength of MTA mixed with CaCl_2_ and 1% methyl cellulose [4], and also microleakage of MTA mixed with 10% CaCl_2_ [7]. To the best of our knowledge, this is the only study that has compared the apical seal of the MTA mixed with three vehicles including saline, 5% CaCl_2_ and KY Jelly under controlled conditions. In the present study it has been decided to simulate the specimen as precise as possible to decrease the interfering variables.

Different techniques have been used for the assessment of microleakage, including fluid filtration technique, electrochemical microleakage test and bacterial microleakage test [2]. The most popular method was linear measurement of tracer (dye or radioisotope) penetration along the root filling. Unfortunately, there is not any precise and reproducible leakage test available so far to meet all requirements [8]. In the present study, however, dye penetration and then clearing technique was used to assess microleakage, which is a simple yet inexpensive method and is commonly being used. Dye penetration and clearing technique is a 3-dimensional method which detects the maximum microleakage in different aspects [9]. It was also tried to use the best clearing method available (10% HCl) in the present study. This method allows us to see through the dental root canal and even to distinguish the accessory canals. Also, to further simulate the clinical situation, the specimens were embedded into a sponge (PDL simulation). Rotary ProFile #40 with a 0.06 taper (diameter=1.36 mm) was used to standardize the specimens based on actual anatomic simulation. The absence of dye penetration in the negative control group and its presence in the positive control group confirms the reliability of the laboratory procedure.

The findings of the present study are consistent with a considerable amount of the literature, mostly confirming the negligible microleakage of the open-apex roots following the application of MTA. To date no study has concentrated on the combination of MTA and KY Jelly. Tang HM *et al.* compared the efficacy of amalgam, MTA, Super-EBA and IRM in apical root sealing. It was shown that MTA is more efficient than amalgam and provides more prolonged sealing compared to Super-EBA [10]. Also the findings of the present study regarding the high sealing potential of the MTA mixed with normal saline is totally consistent with the findings of Torabinejad *et al.* [11] and Nakata [12]. The high sealing ability of MTA in combination with normal saline has been supported by all these studies. Also our study proved the superiority of the combination of MTA with normal saline compared to 5% CaCl_2_ and KY Jelly. Al-Kahtani *et al.* evaluated the microleakage of an orthograde apical plug of mineral trioxide aggregate in permanent teeth with simulated immature apices *in vitro *[3]. Only 5 mm MTA plug group did not show any bacterial leakage in their study. Other groups with different plug thicknesses (*i.e.* 2 and 4 mm plugs of MTA with gutta-percha backfill) showed 100% leakage of *A. Viscous*. In the present study we have used 4 mm MTA layer thickness to achieve the maximum apical seal. 

Hachmeister *et al.* evaluated the sealing and retention characteristics of MTA in an apexification model [13]. It was concluded that MTA is capable of sealing the immature pulpless teeth. This bacterial leakage study revealed that 4 mm MTA plug, with or without prior treatment with calcium hydroxide, is associated with leakage in 13 out of 14 specimens after 10 days. No further leakage, however, was detected during the next 60 days. Also in another group in which MTA was used to obturate the whole canal, no leakage was observed, which was indicative of high sealing potential of MTA mixed with normal saline [13]. In accordance to our result, Daoudi *et al.* compared the sealing ability of Vitrebond cement and MTA and showed less microleakage with MTA compared to that of Vitrebond group in which all specimens had microleakage except for one [14]. All the mentioned studies are supportive of our result on the superior sealing characteristic of the combination of MTA and normal saline [14].

Hong *et al.* compared the apexification of three materials including osteogenic protein- 1, calcium hydroxide, and MTA using flow porometry [2]. They concluded that the addition of 10% CaCl2 to the Portland cement and MTA will significantly decrease the microleakage in one-step apexification. The difference between the findings of the present study and the study by Hong *et al.* may be due to the different assessment methods, CaCl2 content and the test time intervals. In the present study the specimens were placed into dye for a week while they assessed the leakage by flow porometry test after 90 min and 48 h. On the other hand, MTA mixed with 10% CaCl2 showed a significantly higher microleakage after 48 h compared 90 min, which is consistent with our findings. Also in their study, 0.1 g of 10% CaCl2 powder was solved in 3 mL normal saline and then added to MTA powder while in the present study, 5% liquid CaCl2 (solved in normal saline) was mixed with 1g MTA powder with 0.30 mL proportion. The different results of the present study and the study by Hong *et al.* may also be well described by the different proportions of used materials. It seems that more studies on the optimal proportion of MTA and CaCl_2_ with the least microleakage will be necessary. The findings of Martin *et al.* in 2007 are also inconsistent to the findings of the present study [15]. They assessed the apical sealing potential of orthograde MTA plug in an *in vitro* apexification model. They observed a superior apical seal when MTA was used to obturate the whole root canal compared to apical plug group. Apical seal of these two groups, however, was not significantly different after 4 weeks. The interaction of MTA and phosphate buffered saline (PBS), in which the specimens where soaked, may result in sedimentation of apatite which may enhance the apical seal of MTA by time [16, 17]. In the present study, however, the combination of MTA with other vehicles including 5% CaCl_2_ and KY Jelly may have impaired the apical seal of apical plug. Since no other studies have used the same density of used substances, it is not possible to make a logical comparison.

Although the addition of 5% CaCl_2_ and KY Jelly reduces the setting time of MTA to 20-25 min [5], the present study suggests that using vehicles other than saline significantly impairs the sealing ability of the MTA apical plug. This can be attributed to the fact that the sealing potential of MTA is related to its hydrophilic characteristic and its expansion while being mixed with water [1]. MTA, however, may lose its consistency when mixed with 5% CaCl_2_ and KY Jelly, which is confirmed by the decreased compressive strength [5]. Less intermolecular distance of MTA mixed with normal saline compared to that of MTA mixed with 5% CaCl_2_ and KY Jelly may well describe the above-mentioned phenomenon. Ber *et al.* concluded that the compressive strength of the MTA mixed with 2% CaCl_2_ is almost the same as that of MTA mixed with normal saline [4]. On the other hand they have stated that CaCl_2_ in concentrations higher than 2% will impair the characteristics of Portland cement and increases the possibility of drying shrinkage hence decreasing the mixture strength. It may then be concluded that 5% CaCl_2_ will impair the mechanical seal of MTA in open-apex roots.

## Conclusion

Within the limitations of this *in vitro* study it may be concluded that among saline, 5% CaCl_2_, and KY Jelly as three different vehicles for ProRoot MTA apical plug, saline is associated with the least microleakage in canals with open apices.
